# DUSP3/VHR is a pro-angiogenic atypical dual-specificity phosphatase

**DOI:** 10.1186/1476-4598-13-108

**Published:** 2014-05-15

**Authors:** Mathieu Amand, Charlotte Erpicum, Khalid Bajou, Fabio Cerignoli, Silvia Blacher, Maud Martin, Franck Dequiedt, Pierre Drion, Pratibha Singh, Tinatin Zurashvili, Maud Vandereyken, Lucia Musumeci, Tomas Mustelin, Michel Moutschen, Christine Gilles, Agnes Noel, Souad Rahmouni

**Affiliations:** 1 Immunology and Infectious Diseases, GIGA-Signal Transduction, University of Liège, Liège 4000, Belgium; 2 Laboratory of Tumor and Developmental Biology, GIGA-Cancer, University of Liège, Liège, Belgium; 3 GIGA-Pre-Clinical Tumor Model Core-Facility, University of Liège, Liège 4000, Belgium; 4 Signal Transduction program, Sanford-Burnham Medical Research Institute, La Jolla, CA 92037, USA; 5 Laboratory of Protein Signaling and Interactions, GIGA-Signal Transduction, University of Liège, Liège 4000, Belgium; 6 Animal Facility (B23), University of Liège, Liège 4000, Belgium; 7 Present address: MedImmune, One Medimmune Way, Gaithersburg, MD 20878, USA

**Keywords:** DUSP3/VHR, *Dusp3*-knockout mice, Angiogenesis, Endothelial cells, Dual-specificity phosphatase

## Abstract

**Background:**

DUSP3 phosphatase, also known as ***V*** *accinia*-**H**1 **R**elated (VHR) phosphatase, encoded by *DUSP3*/*Dusp3* gene, is a relatively small member of the dual-specificity protein phosphatases. *In vitro* studies showed that DUSP3 is a negative regulator of ERK and JNK pathways in several cell lines. On the other hand, DUSP3 is implicated in human cancer. It has been alternatively described as having tumor suppressive and oncogenic properties. Thus, the available data suggest that DUSP3 plays complex and contradictory roles in tumorigenesis that could be cell type-dependent. Since most of these studies were performed using recombinant proteins or in cell-transfection based assays, the physiological function of DUSP3 has remained elusive.

**Results:**

Using immunohistochemistry on human cervical sections, we observed a strong expression of DUSP3 in endothelial cells (EC) suggesting a contribution for this phosphatase to EC functions. DUSP3 downregulation, using RNA interference, in human EC reduced significantly *in vitro* tube formation on Matrigel and spheroid angiogenic sprouting. However, this defect was not associated with an altered phosphorylation of the documented *in vitro* DUSP3 substrates, ERK1/2, JNK1/2 and EGFR but was associated with an increased PKC phosphorylation. To investigate the physiological function of DUSP3, we generated *Dusp3*-deficient mice by homologous recombination. The obtained DUSP3^−/−^ mice were healthy, fertile, with no spontaneous phenotype and no vascular defect. However, DUSP3 deficiency prevented neo-vascularization of transplanted b-FGF containing Matrigel and LLC xenograft tumors as evidenced by hemoglobin (Hb) and FITC-dextran quantifications. Furthermore, we found that DUSP3 is required for b-FGF-induced microvessel outgrowth in the aortic ring assay.

**Conclusions:**

All together, our data identify DUSP3 as a new important player in angiogenesis.

## Background

The human genome harbors 104 genes encoding for cysteine-based (Cys-based) phosphatases classified into three classes on the basis of their amino acid sequences and catalytic domains [1]. Dual-specificity phosphatases (DUSPs) or *Vaccinia*-H1-like (VH1-Like) enzymes represent the largest group of class I of Cys-based motif phosphatases and is represented by 61 members with diverse substrates specificity ranging from mRNA to inositol phospholipids, p-Ser/p-Thr and p-Tyr. Among these 61 phosphatases, 11 are specific for the MAPKs ERK, JNK and p38 and are known as the typical DUSPs or MAPK specific phosphatases (MKPs). The second group of the VH1-like phosphatases is known as the atypical DUSPs (A-DUSPs) represented by 19 small enzymes (with less than 250aa) and are poorly characterized (reviewed in [2]). Considering the important role of MAPKs in the regulation of different cellular functions and their involvement in different human diseases including cancer [3-5], the activation as well as inhibition processes of these serine/threonine kinase family has been well characterized. Therefore, among all DUSPs, the MKPs have been the most characterized *in vitro* and *in vivo*. The expression of several phosphatases belonging to this group is altered in human cancer (reviewed in [6]).

DUSP3, also called ***V*** *accinia***H**1-**R**elated (VHR), is the founding member of the dual-specificity protein phosphatases group. It consists of a 185 amino acids (Mr 21 kDa) catalytic domain but no apparent targeting domain or docking site and is encoded by *DUSP3/Dusp3* gene [7]. The crystal structure of DUSP3 has been solved and shows a shallow active site allowing DUSP3 to act on both pTyr and pThr in its substrates [8]. DUSP3 has been reported to dephosphorylate the MAPKs ERK and JNK, but not p38 [7-9]. More recently, EGFR and ErbB2 were reported as direct new substrates for this phosphatase in a non-small cell lung cancer cell line NSCLC [10]. Unlike many other MKPs, DUSP3 expression is not induced in response to activation of MAPKs, but is regulated during cell cycle progression [11,12]. In a previous study, we have shown that in HeLa cells, the knockdown of endogenous DUSP3 using RNA interference induces cell cycle arrest at G1/S and G2/M phases and is accompanied by the hyperactivation of ERK1/2 and JNK1/2 [11,12]. In line with this finding, DUSP3 was found up-regulated in human cancers and in several cancer cell lines. Indeed, we reported that DUSP3 is highly expressed in cervical carcinomas and in several cervix cancer cell lines [13]. This phosphatase is also highly expressed in human prostate cancer and in the LNCaP human prostate adenocarcinoma cell line [14]. On the other hand, recent reports showed that DUSP3 is downregulated in NSCLC and when overexpressed in these cells, it leads to decreased cell proliferation and reduced tumor growth in a xenograft mouse model [10]. In line with these findings, Min Gyu Lee’s group reported recently that DUSP3 downregulation in NSCLC tumors, when correlated with high levels of the histone H3 lysine 36 (H3K36) demethylase, KDM2A, is associated with poor prognosis for the patients [15]. In the same study, the authors demonstrated that KDM2A activates ERK1/2 through epigenetic repression of *DUSP3* expression via demethylation by H3K36 at the *DUSP3* locus. DUSP3 has also been found downregulated in breast carcinomas [16]. These studies clearly suggest that DUSP3 plays complex and contradictory roles in tumorigenesis that could be cell type-dependent. However, most of these studies were performed either *in vitro*, using recombinant proteins, or in cell lines, using transient overexpression or siRNA knockdown. Furthermore, all these studies were focused on tumor cells without taking into account the host cells. Therefore, the physiological function of DUSP3 is unknown.

We report herein that DUSP3 is highly expressed in endothelial cells (EC), depletion of which causes an inhibition of EC *in vitro* tubulogenesis. To investigate the physiological functions of DUSP3, we generated a new mutant mouse strain deficient for *Dusp3* gene. The obtained DUSP3-deficient mice were viable and had no apparent phenotype or spontaneous pathology, suggesting that these mice could be useful to study DUSP3’s role in different pathological conditions. Indeed, by applying different *in vivo*, *ex vivo* and *in vitro* models, we provide evidence that DUSP3 plays an important and non-redundant role in angiogenesis.

## Results

### DUSP3 is highly expressed in human endothelial cells and its expression is required for *in vitro* tubulogenesis

**Figure 1:**
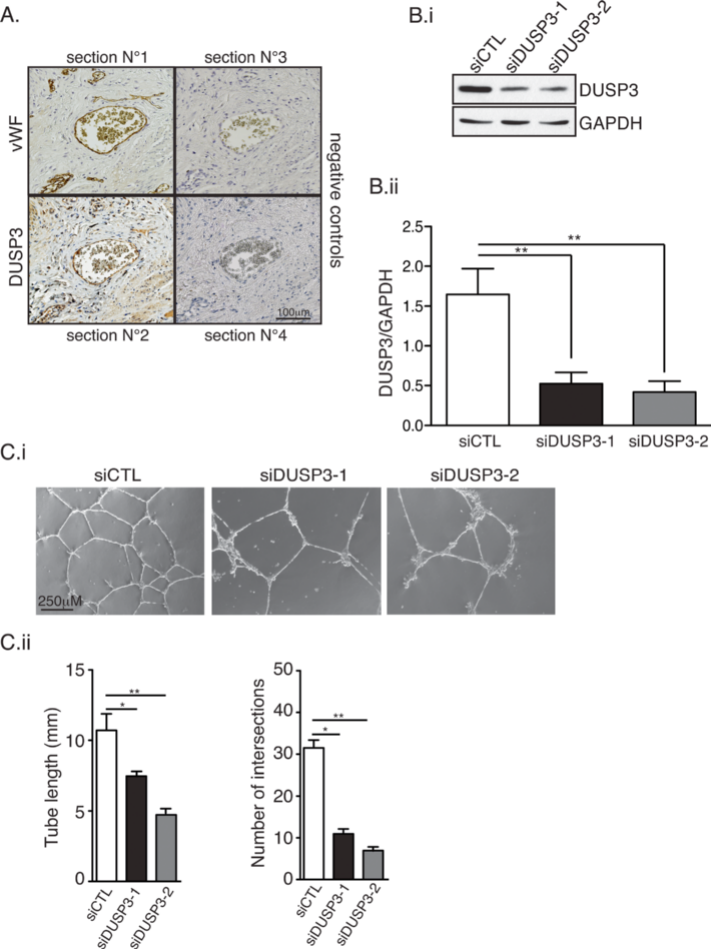
**DUSP3 is highly expressed in endothelial cells and its dowregulation inhibits in vitro tubulogenesis. (A)** Immunohistochemistry of DUSP3 and Von Willbrand Factor (vWF) on paraffin embedded 4 μm serial sections of human cervix biopsies. Section 1 was stained with anti-vWF antibody and section 2 with anti-DUSP3 antibody. Sections 3 and 4 were stained with the secondary antibodies used to reveal vWF and DUSP3 staining respectively. **(B)** HUVEC cells were transfected with non-targeting siRNA (siCTL) or with DUSP3 targeting siRNA (siDUSP3-1 and siDUSP3-2). Efficiency of DUSP3 downregulation was measured at protein level using western blot 72 h after cell transfection. **(Bi)** Equal amount of proteins were resolved by SDS-PAGE, and western blot was performed using anti-DUSP3 antibody or anti-GAPDH as loading control. **(Bii)** Quantification and statistical analysis of DUSP3 protein expression in siCTL, siDUSP3-1 and siDUSP3-2 transfection conditions represented as a ratio of DUSP3 on GAPDH. **(Ci)** Phase contrast microscopy of siCTL, siDUSP3-1 and siDUSP3-2 transfected HUVECs seeded on pre-solidified Matrigel for 16 hours. **(Cii)** Quantitative analysis of the experiment shown in **(Ci)** obtained by measuring the tube lengths (left panel) and number of intersections (right panel) from 10 fields. *, P < 0,05 and **, P < 0.01.

During our previous study investigating the role of DUSP3 in human cervical cancer [13], we noticed that all the blood vessel walls present in the tissue sections were highly immunoreactive to anti-DUSP3 antibody, suggesting that DUSP3 is highly expressed in endothelial and/or smooth muscle cells, the 2 major blood vessels cell components. To verify this hypothesis, we stained paraffin embedded 4 μm serial sections of human cervix biopsies with anti-DUSP3 or anti-Von Willebrand Factor (vWF) antibodies. As shown in Figure 1A, endothelial cells, identified based on the vWF staining in section 1, were also positively stained with anti-DUSP3 antibody in section 2, confirming DUSP3 high expression in EC. To assess the role of DUSP3 in EC, we downregulated its expression in the primary Human Umbilical Vein Endothelial cells (HUVEC) using DUSP3 targeting siRNA and conducted a tube formation assay on Matrigel. Cells were transfected with non-targeting siRNA (siCTL) or with DUSP3 targeting siRNAs (siDUSP3-1 and siDUSP3-2). The efficacy of the two different DUSP3 targeting siRNA was demonstrated by the significant decrease of DUSP3 protein levels (Figure 1B). 72 hours after transfection, equal cell numbers were seeded in a 24-well plate on a layer of pre-solidified Matrigel. After 24 h, the tube networks were visualized under phase contrast microscope and photographed (Figure 1Ci). Tube network were quantified by measuring total tube length and number of tubes intersections. DUSP3 downregulation induced a significant decrease in tubulogenesis (Figure 1C) as quantified by a significant reduction of network lengths and number of tube intersections (Figure 1Cii) in both DUSP3 targeting siRNA conditions compared to the siCTL condition.

### Downregulation of DUSP3 inhibits *in vitro* angiogenic sprouting

**Figure 2:**
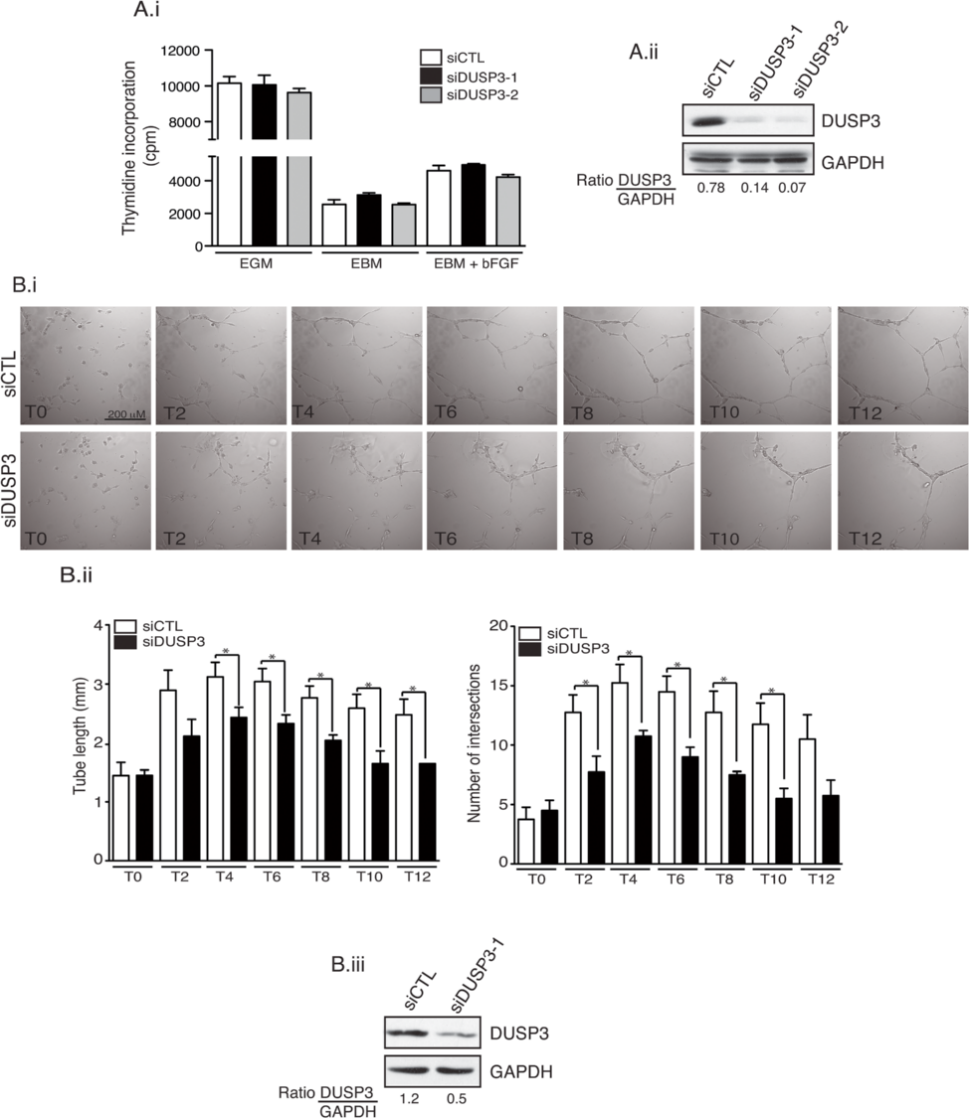
**DUSP3 downregulation affects HUVEC cell angiogenic sprouting but does not affect proliferation. (A)** 72 hours after transfection with siCTL, siDUSP3-1 or siDUSP3-2, HUVECs were trypsinized and 7.5 × 10^3^ from each transfection condition were plated (in triplicate) and cultured for an additional 24 hours. ^3^H-Thymidine was added for the last 4 h before cell harvesting. Radioactivity was counted using a scintillation analyzer. **(Ai)** Statistical analysis from three independent experiments. Data are reported as mean ± SEM. **(Aii)** Western blot analysis using anti-DUSP3 antibody and anti-GAPDH of one representative experiment showing DUSP3 depletion after siDUSP3-1 and siDUSP3-2 transfection. Quantification of DUSP3 protein expression in siCTL, siDUSP3-1 and siDUSP3-2 transfection conditions are shown as a ratio of densitometry values of DUSP3 on GAPDH bands. **(Bi)** Snapshots from phase contrast time-lapse movies of tube formation assay of HUVECs transfected with siCTL or with siDUSP3 and seeded on Matrigel solidified matrix (Additional files 1 and 2). **(Bii)** Quantitative analysis of the snapshots shown in **(Bi)** obtained by measuring the tube lengths (left panel) and number of intersections (right panel) from 10 fields. **(Biii)** Western blot and quantification of DUSP3 protein expression in siCTL and siDUSP3-1 transfection conditions represented as a ratio of DUSP3 on GAPDH.

**Figure 3:**
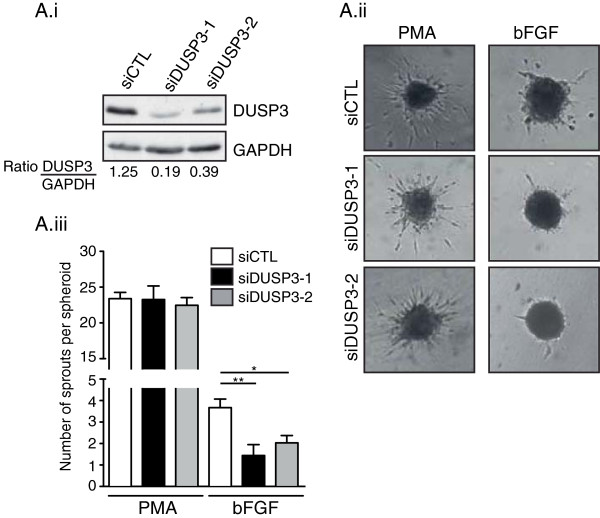
**DUSP3 downregulation affects HUVEC spheroids sprouting. (Ai)** Western blot and quantification of DUSP3 protein expression in siCTL, siDUSP3-1 and siDUSP3-2 transfection conditions represented as a ratio of DUSP3 on GAPDH. **(Aii)** Representative images of spheroid sprouting assay performed with endothelial cells as indicated with siCTL, siDUSP3-1 and siDUSP3-2 in the presence of PMA (75 ng/mL) or b-FGF (10 ng/mL). **(Aiii)** The mean cumulative number of sprouts per spheroid was assessed after 48 hours. Results are presented as mean ± SEM. *, P < 0,5; **, P < 0,01.

We previously found that DUSP3 depletion halted HeLa cell proliferation [11]. We also reported that DUSP3 inhibition using small inhibitors blocked HeLa and Caski cell proliferation [17]. Therefore, we postulated that the decreased tubulogenesis in DUSP3 depleted EC could be due to a defect in cellular proliferation. To investigate this hypothesis, we measured HUVEC cells proliferation 72 hours post transfection with the different siRNAs. HUVECs proliferation, as measured by thymidine incorporation (cpm), was not affected by DUSP3-depletion in any of the conditions analyzed, namely in the EGM rich medium, in the EBM minimum medium and in the EBM b-FGF growth factor (100 ng/mL)-supplemented medium (Figure 2Ai). Efficiency of DUSP3 depletion is shown in Figure 2Aii. We then hypothesized that the observed decrease of tube formation in DUSP3-depleted conditions could be due to a defect in endothelial sprouting. Thus, we performed time-lapse under confocal microscopy. 72 hours after HUVECs transfection, equal numbers of cells were seeded on pre-solidified-Matrigel in chamber slides. Chambers were immediately transferred on the x-y-z stage of Nikon microscope equipped with a cell culture chamber at 37°C and 5% CO_2_. Images were acquired every 10 min for 12 h. As demonstrated on the Additional files 1 and 2, HUVECs transfected with siDUSP3 failed to form stable sprouts while upon siCTL transfection, cells formed homogenous and vigorous sprouts (Additional files 1 and 2 and Figure 2B). The quantification of sprouting showed a significant decrease of tube length and number of intersections in the siDUSP3 condition compared to siCTL condition at all time points analyzed during the acquisition time period (Figure 2Bii). A representative western blot showing the efficiency of DUSP3 depletion for these experiments is shown in Figure 2Biii. These findings were further confirmed using the spheroid-sprouting assay. Indeed, DUSP3 silencing, as evidenced by DUSP3 downregulation (Figure 3Ai), blocked significantly the angiogenic sprouting of HUVECs upon stimulation with b-FGF as demonstrated by the decline of sprouts numbers per spheroid. However, when cells were stimulated with PMA, as a positive control, sprouting of HUVECs was equally induced in siCTL and siDUSP3 conditions (Figure 3Aii-Aiii). These data suggest that DUSP3 contributes to growth factors-induced angiogenic sprouting.

### DUSP3 depletion did not affect the MAPKs and EGFR but affected PKC phosphorylation in HUVECs

**Figure 4:**
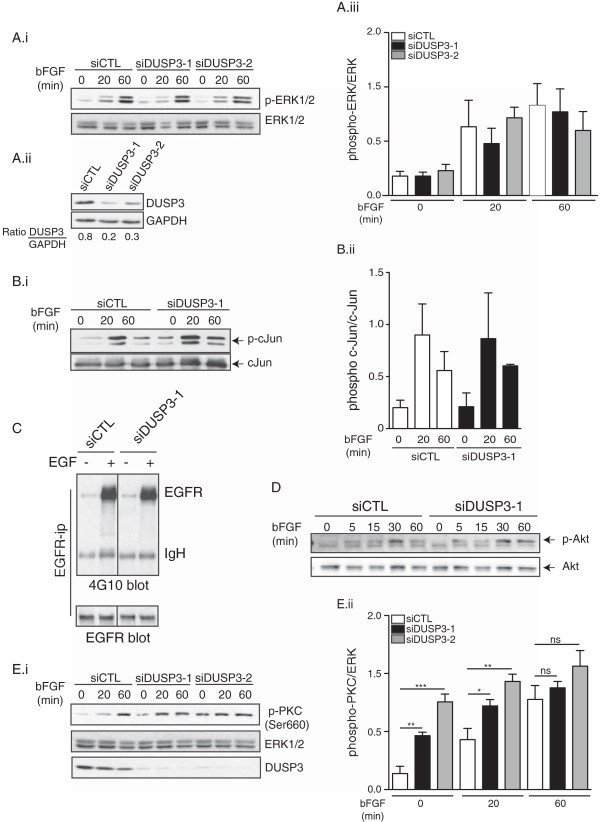
**DUSP3 depletion affects PKC activation but is dispensable for ERK1/2, JNK and EGFR in HUVEC cells.** HUVECs were transfected with non-targeting siRNA (siCTL) or with DUSP3 targeting siRNA (siDUSP3-1 and siDUSP3-2). 24 h before stimulation, cells were washed and let to rest overnight in 2% serum containing medium. Cells were then activated with b-FGF (10 ng/mL) for the indicated time points theb lysed. Cell lysates were resolved on SDS-PAGE and immuno-reacted with **(Ai)** anti-phospho-ERK1/2 (Thr202/Tyr204) and ERK as an internal loading control, **(Aii)** anti-DUSP3 and anti-GAPDH **(Aiii)** Quantification of the phosphorylation levels or ERK was determined by densitometric analysis and is shown as a ratio of pERK/ERK. Results are presented as mean ± SEM and are representative of 3 independent experiments. **(B)** SAPK/JNK kinase assay. JNK was immunoprecipitated from siCTL and siDUSP3 transfected cell lysates. After transfer of the JNK immunoprecipitates, nitrocellulose membranes were immuno-reacted with anti-phospho-c-Jun and anti-c-Jun antibodies **(Bi)**. Quantification of the phosphorylation levels or JNK substrate, c-Jun, was determined by densitometric analysis and is shown as a ratio of p-c-Jun/c-Jun **(Bii)**. (**C**) EGFR phosphorylation. EGFR was immunoprecipitated from non-stimulated and EGF (100 ng/ml) stimulated HUVECs transfected with siCTL or with siDUSP3. Immunoprecipitates were immunoreacted with anti-phosphotyrosine antibody 4G10. Membranes were stripped and re-bloted with anti-EGFR antibody. **(D)** Western blot for p-Akt and Akt on cell lysate from FGF stimulated siCTL and siDUSP3 transfected conditions. **(Ei)** Western blot for phospho-PKC (Ser660) on cell lysates from FGF stimulated cells in the conditions indicated. ERK was used as a loading control. **(Eii)** Quantification of the phosphorylation levels or PKC was determined by densitometric analysis and is shown as a ratio of phospho-PKC/ERK. Results are presented as mean ± SEM and are representative of 4 independent experiments. *p < 0,05; **p < 0.01; ***p < 0,001.

*In vitro* DUSP3 most studied substrates are the mitogen-activated protein kinases (MAPKs) ERK1/2 and JNK, but not p38 [7-9,18]. In a previous study, we reported that DUSP3 downregulation in HeLa cells halts cell proliferation and associates with the ERK1/2 and JNK1/2 hyperphosphorylation [11]. Therefore, we investigated if in EC, DUSP3 depletion could lead to a modification of the kinetic and/or the magnitude of ERK1/2 and or JNK1/2 activation. 48 h after HUVECs transfection using DUSP3 targeting siRNAs or siCTL, cells were washed and incubated for 24 h in 2% serum containing-medium. Cells were next washed and activated with 10 ng/ml of b-FGF for 20 and 60 min at 37°C. Cells were then lysed and western blots were performed using phosphospecific antibodies against ERK1/2 activated forms. On the contrary to our previous findings in HeLa cells, we found that DUSP3 depletion in HUVEC cells did not affect ERK1/2 activation kinetic and magnitude (Figure 4Ai and Aii). To assess the JNK activity in the absence of DUSP3, we performed a SAPK/JNK kinase assay by immunoprecipitating endogenous phospho-SAPK/JNK from resting or b-FGF activated siCTL and siDUSP3 transfected cells. The activity of JNK was revealed by incubating phospho-SAPK/JNK immunoprecipitates with recombinant c-Jun, the JNK downstream target. SAPK/JNK-induced recombinant c-Jun phosphorylation was measured by a quantitative immunoblotting using phospho-c-Jun (Ser63) and c-Jun antibodies. As demonstrated by the results shown in Figure 4B, DUSP3 downregulation did not impact the kinetic and/or magnitude of c-Jun phosphorylation suggesting that JNK activity is not affected by DUSP3 depletion in EC.

A previous study has also reported that DUSP3 has a minimal effect on MAPK phosphorylation but rather target directly EGFR in non-small cell lung cancer cell line [10]. To investigate if this is also the case in endothelial cells, siCTL and siDUSP3 transfected HUVECs were activated using EGF (100 ng/ml). EGFR was then immunoprecipitated and immunoreacted with 4G10 anti-phosphotyrosine antibody. As shown in Figure 4C, DUSP3 depletion did not affect EGFR tyrosine phosphorylation. All together, these results suggest that DUSP3 does not target MAPKs and EGFR in EC.

The fact that DUSP3 dowregulation in HUVECs does not affect cell proliferation and ERK1/2 activation suggests that DUSP3 is dispensable for the b-FGF-induced cell proliferation. Since FGF signaling is also involved in prosurvival via the activation of PI3k/Akt pathway, we investigated if DUSP3 deficiency could lead to cell death or could impact Akt activation. We found that DUSP3 depletion in HUVECs was not associated with increased cell death as measured by AnnexinV-PI (data not shown). Consistent with this finding, DUSP3 downregulation did not affect b-FGF-induced Akt phosphorylation (Figure 4D). FGF plays also a crucial role in cell migration and angiogenesis. This effect is mediated through the PI3K and PLCγ/PKC activation pathways [19]. Therefore, we hypothesized that DUSP3 affects the PLCγ/PKC activation pathway in our model. To investigate this hypothesis, we subjected the resting and b-FGF activated HUVECs lysates to immunoblot using phospho-PKC (Ser660) and found that PKC was significantly hyper-phosphorylated at basal levels in the absence of DUSP3 compared to the siCTL condition. The activation with b-FGF increased further the phosphorylation of PKC in all conditions. However, in the DUSP3 downregulated conditions, the phosphorylation of PKC plateaued earlier (at 20 min after stimulation) than in siCTL (Figure 4E). These results suggest that DUSP3-depletion-associated sprouting defect in HUVECs could be the consequence of a defect in the PKC activation pathway.

### DUSP3-deficient mice are healthy and do not exhibit any spontaneous phenotype

**Figure 5:**
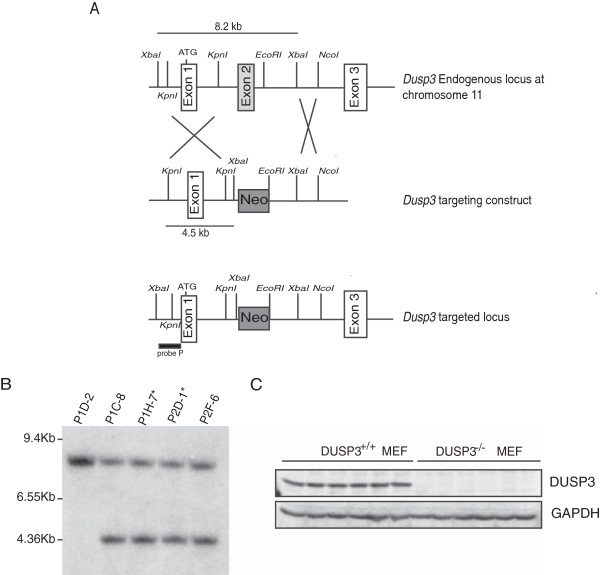
**Dusp3 deficient mice generation by targeted homologous recombination. (A)** Schematic diagram showing part of the *Dusp3* gene locus, the targeted *Dusp3* construct and the resulting targeted allele. Recombination events are indicated by open white boxes and show the replacement of a 8.2 kb *Dusp3* genomic fragment containing exon II by the pPNT-Neo cassette. **(B)** Southern blot analysis of ES cells genomic DNA following digestion with *XbaI* using a 5’ external Probe P as indicated in **(A)**. The autoradiography revealed the 8.2 kb (wild-type) and 4.5 kb (targeted) fragments. The stars represent the ES cell lines used for microinjection of mouse blastocysts. **(C)** Western blot analysis of DUSP3 protein expression in MEF cell extracts from 6 *Dusp3*^+/+^ and 6 *Dusp3*^−/−^ mice. GAPDH was used as an internal control.

To gain insights into the function of DUSP3 in EC under physiological conditions, we generated *Dusp3*-deficient mice by targeted homologous recombination. The *Dusp3* gene was disrupted in 129/SvJ murine embryonic stem (ES) cells by the replacement of exon II with a *neo* gene expression cassette (Figure 5A). Four ES clones, containing the targeted disrupted allele, were obtained and injected into blastocysts of C57BL/6 J mice. Germline transmission was obtained from 2 independent ES cell clones. Southern blot analysis confirmed the presence of the disrupted exon (Figure 5B). Heterozygous mice did not show morphological abnormalities and were bred to obtain mice homozygous for the disrupted allele. *Dusp3*^−/−^ mice showed no detectable protein immunoreactivity with anti-DUSP3 antibody in protein extracts from mouse embryonic fibroblasts (MEFs) compared to wild-type mice (Figure 5C). These mice were viable, fertile, developed normally and had no apparent or spontaneous pathology. These finding suggest that DUSP3 is dispensable for embryogenesis, adult mice development and homeostasis. A second alternative could be that another DUSP is compensating for DUSP3 deficiency.

### DUSP3 deficiency affects *in vivo* and *ex vivo* angiogenesis

**Figure 6:**
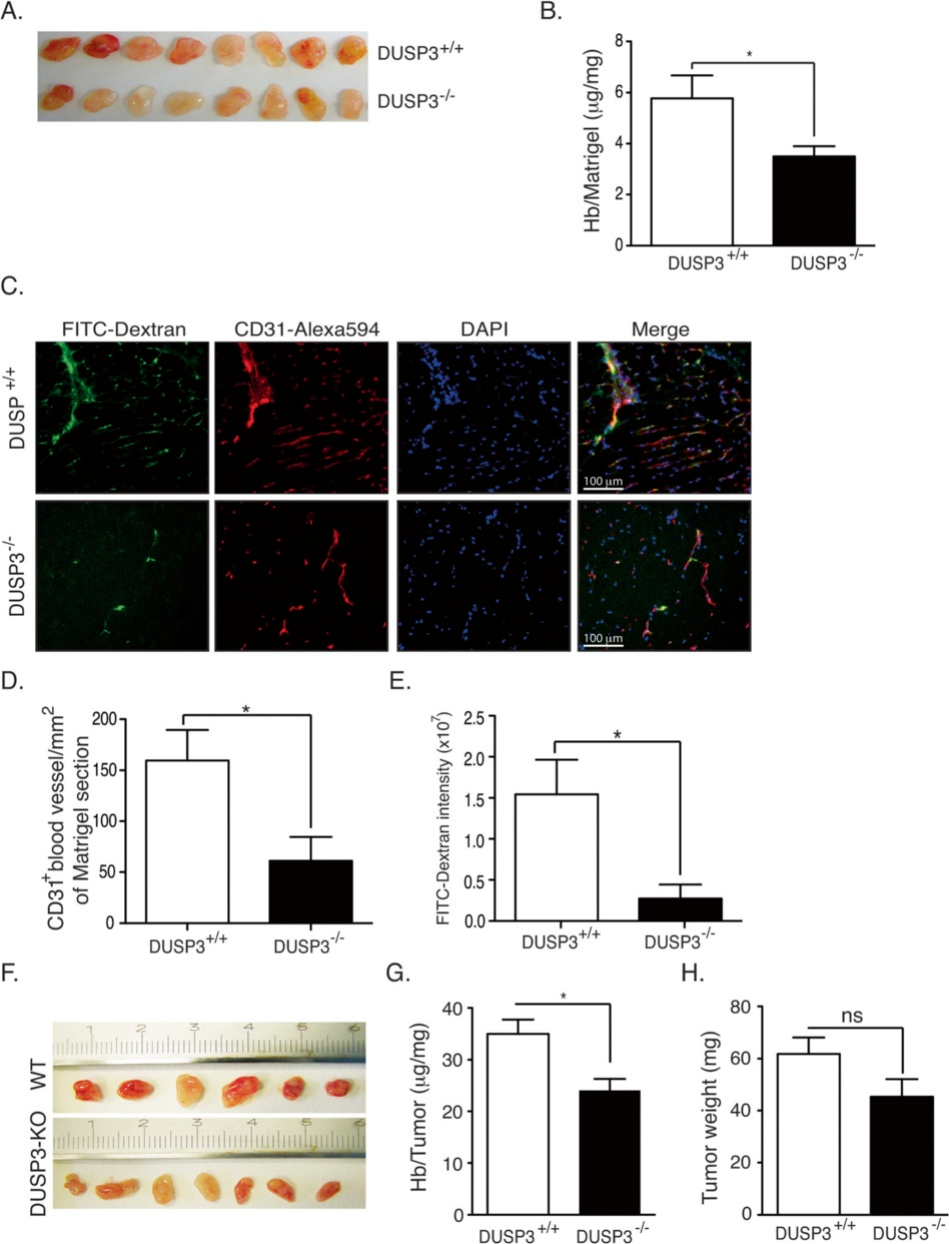
**DUSP3 deficiency affects in vivo angiogenesis. (A-E)** DUSP3^+/+^ and DUSP3^−/−^ mice were injected subcutaneously in the flanks with 0.5 mL of Matrigel together with human b-FGF (250 ng/mL) and Heparin (0,0138 μg/mL). Ten days after injection, mice were sacrificed and Matrigels were removed. Representative photographs are shown in **(A)** from eight mice from each group. **(B)** Quantification of angiogenesis within the Matrigel plugs was achieved by measuring hemoglobin (Hb) concentration in the Matrigel homogenates and was reported as mg of measured Hb per mg of Matrigel. Results are presented as means ± SEM, *n* =15 in each group. **(C)** FITC-dextran was i.v. injected to DUSP3^+/+^ and DUSP3^−/−^ mice 5 min before removal of subcutaneously implanted Matrigel plugs. Frozen sections were stained using Alexa 594 conjugated anti-CD31 and DAPI and visualized using fluorescent microscope. Representative micrographs of FITC-Dextran (green), CD31 (red), DAPI (blue) stainings and a merge of all are shown. **(D-E)** Quantification of CD31^+^ cells in blood vessel sections per mm^2^ of Matrigel section **(D)** and FITC dextran intensity (arbitrary units) in Matrigel sections **(E)** from DUSP3^+/+^ and DUSP3^−/−^ mice. Results are presented as means ± SEM, *n* = 10 in each group. Data are presented as mean ± SEM from 3 independent experiments (n = 15). **(F-H)** 10^6^ LLC cells were subcutaneously injected in the flank of DUSP3^+/+^ and DUSP3^−/−^ female mice. 7 days later, tumors were removed and homogenized for Hb measurement. Representative photographs of the tumors are shown in **(F)** from six WT and seven DUSP3-KO mice. **(G)** Hb measurement in the tumors homogenates shown in **F**. **(H)** Weights of the tumors retrieved from the mice. Data are presented as mean ± SEM. *p < 0.05 (t-student test).

The altered expression of DUSP3 in several human cancers [13,14,16] and the newly discovered role of DUSP3 in EC tubulogenesis prompted us to investigate if *in vivo* DUSP3 deficiency could also lead to a decrease in neovascularization and angiogenesis. We have used three well established models of angiogenesis, previously validated to assess the function of different metalloproteinases in angiogenesis [20-22]. To perform our comparative studies and assess the angiogenic response to b-FGF in DUSP3^+/+^ and DUSP3^−/−^ mice, 500 μL of Matrigel containing human b-FGF (250 ng/mL) and Heparin (0.0138 mg/mL) were injected subcutaneously to the two flanks of DUSP3^+/+^ and DUSP3^−/−^ mice (n = 15 mice in each group). Quantification of plugs vascularization was performed 10 days after injection. As shown in Figure 6A, plugs retrieved from DUSP3^−/−^ mice were clearly less vascularized compared to the ones harvested from DUSP3^+/+^ mice. This was confirmed after homogenization of the Matrigel plugs and measurement of their hemoglobin (Hb) content (Figure 6B). Indeed, Matrigel from the DUSP3^−/−^ mice showed more then 40% decrease of Hb compared to the plugs from DUSP3^+/+^ mice. To further confirm these findings, in a separated experiment, mice were injected with FITC-dextran 5 min prior to Matrigel plugs removal. FITC-dextran fluorescence, together with CD31 staining was next visualized under epifluorescence microscope and fluorescence was quantified using Imaris software. Matrigels retrieved from DUSP3^−/−^ mice showed a minimal vascularization as demonstrated by the level of FITC-dextran fluorescence intensity (Figure 6C-D) and by the CD31^+^ staining (Figure 6C and E).

The decrease of Matrigel vascularization in DUSP3^−/−^ mice was further confirmed by the significant decrease of representative endothelial cells transcripts, such as *Pcam1* (CD31) and *Cdh5* (VE-Cad/Cd144), and pericytes transcripts, such as *Acta2* (aSMA) and *Pdgfrb* (CD140b) in the Matrigel plugs retrieved from DUSP3^−/−^ compared to the ones from DUSP3^+/+^ mice (Table 1).

**Table 1:** **Representative gene expression profile of different cell populations **[23]** in Matrigel plugs from Dusp3-knockout versus WT**

**Gene name**	**Alternative names**	**Ratio *Dusp3*-KO/WT**	***P value***
***Endothelial cells***			
*Pecam1*	Cd31; PECAM-1	0.52	0.045*
*Mcam*	CD146; CD149	0.46	0.060
*Tie1*	TIE; tie-1	0.37	0.059
*Cdh5*	VECD; Cd144; VE-Cad;	0.35	0.038*
***Pericytes***			
*Cspg4*	AN2; NG2	0.76	0.25
*Pdgfrb*	CD140b; PDGFR-1	0.66	0.047*
*Acta2*	a-SMA; alphaSMA	0.29	0.034*
***Lymphoid and Myeloid markers***			
*Csf1r*	CD115; CSF-1R	1.2	0.58
*Itgam*	MAC1; Cd11b; CD11b/CD18	1.1	0.31
*Klrb1c*	CD161; NK1.1	1.1	0.85
*Ly6a/e*	Sca1; Ly-6A.2	1.1	0.56
*Ly6c1*	Ly6c	1.1	0.56
*Emr1*	F4/80	1.0	0.95
*Lgals3*	Mac-2	1.0	0.58
*Csf1r*	CD115; CSF-1R	1.0	0.97

Data presented are extracted from a genome-wide microarray dataset obtained using Illumina’s multi sample format Mouse WG-6 V2 BeadChip. Data are presented as the ratio of the average values obtained from 2 pools of 3 *Dusp3*-knockout each on 2 pools of 3 WT mice each and the corresponding *p-value*. (*p < 0.05 determined using unpaired student’s *t* test).

We next investigated the angiogenic role of DUSP3 in the context of tumor development by using a rapid tumor-induced model. Mice were subcutaneously injected with 10^6^ of Lung Lewis Carcinomas cells (LLC) and tumors were removed 7 days later. As shown if Figure 6F, the Hb content of the homogenized tumor mass from the DUSP3^−/−^ mice was reduced by 30% compared to the homogenates from the DUSP3^+/+^ mice (Figure 6F-G). LLC tumors weights were reduced slightly but not significantly in the DUSP3^−/−^ compared to DUSP3^+/+^ mice (Figure 6F and H). These results demonstrate that the tumor-induced angiogenic response is defective in mutant mice.

**Figure 7:**
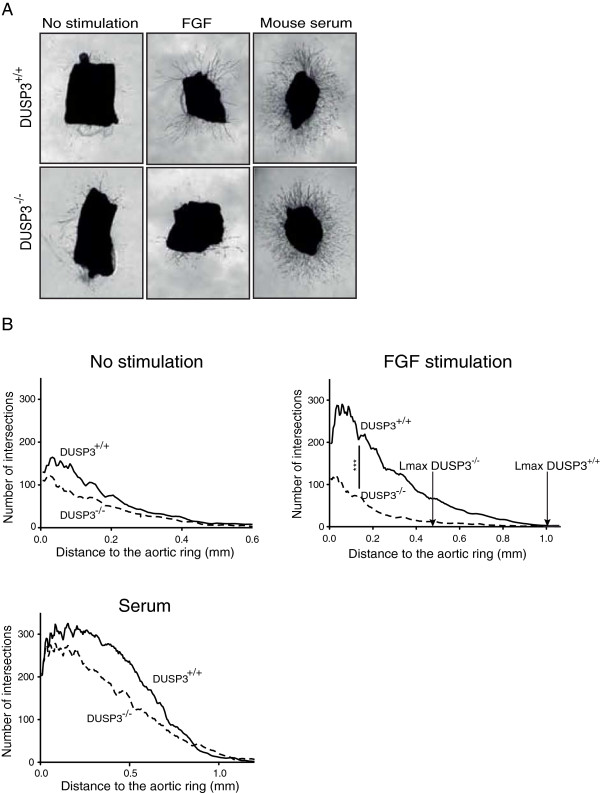
**Ex-vivo microvasculature outgrowth from DUSP3**^**+/+ **^**and DUSP3**^**−/− **^**mice aortic rings. (A)** Phase contrast micrographs of thoracic aortas from 17 weeks old DUSP3^+/+^ and DUSP3^−/−^ mice grown for 9 days in collagen additive-free (no stimulation), in 2.5% serum containing collagen gels or in 20 ng/mL of b-FGF-supplemented collagen gels. Magnification ×25. **(B)** Computerized quantification of number of microvessel intersections and maximal length of vessels from culture conditions shown in A. X axis represents the length of the aortic microvessel outgrowth and Y-axis represents the number of intersections of the microvessels. The arrows in the b-FGF stimulated conditions indicates the maximal vessel growth, Lmax (mm) for DUSP3^−/−^ and for DUSP3^+/+^ aortas. ***p <0.001 (t-student test).

To further investigate the contribution of DUSP3 in neovessel formation, aortic explants issued from DUSP3^+/+^ and DUSP3^−/−^ mice were embedded in three dimensional type I collagen gel in non-complemented medium, autologous serum complemented or in b-FGF complemented medium (Figure 7A). The microvessel outgrowth were quantified by determining the number of intersections in function to the distance to the aortic ring as previously reported [24]. As evidenced by Figure 7, sprout density was significantly reduced (p < 0,001) in the aortas derived from DUSP3^−/−^ mice when stimulated with b-FGF growth factor (Figure 7A-B). Furthermore, the measured length achieved by vessels was reduced in the absence of DUSP3 upon stimulation with b-FGF. Indeed, maximal vessel growth for DUSP3^−/−^ aortas was: Lmax (mm) = 0.48 ± 0.23 and for DUSP3^+/+^ aortas: Lmax = 1.195 ± 0.11 (p Value = 0.021). Differences were not statistically significant neither in the non-stimulated nor in the serum-stimulated conditions (Figure 7A-B).

All together, these results suggest that, *in vivo*, DUSP3 plays a key role in neoangiogenesis.

## Discussion

The physiological function and possible involvement of the A-DUSP family members in cancer is largely unknown. The lack of knockout mice for A-DUSPs is probably one of the major limitations in the determination of the physiological function of these phosphatases. So far, out of 19 A-DUSPs, only 3 were disrupted in mice, STYX [25], DUSP14 [26] and laforin [27]. However, the role of these phosphatases in cancer and angiogenesis were not investigated in these mutant mice. Thus, the physiological function of A-DUSPs in cancer and angiogenesis is still unknown.

We report here the generation of a new mouse strain lacking *Dusp3* gene, encoding for the atypical dual specificity phosphatase DUSP3. The mutant DUSP3^−/−^ mice develop normally and do not have any spontaneous evident pathology, making them a good *in vivo* tool to investigate the role of DUSP3 in different diseases. By applying different *in vivo* and *ex vivo* models to these knockout mice, we provide evidence for a new physiological role of DUSP3 in neovascularization. We also report that DUSP3 is highly expressed in human endothelial cells and demonstrate its essential role for *in vitro* primary human endothelial cell angiogenic sprouting function.

The fact that DUSP3-deficient mice are born displaying no vascular defects under normal conditions could be explained by a redundant function of DUSP3 shared with other DUSPs. Indeed, several DUSPs have overlapping substrates specificity, especially among MAPKs. This makes it difficult to assign a specific physiological role for a specific DUSP in a specific tissue. It is conceivable that conditional knockout mice lacking DUSP3 only in the endothelial cells may display a vascular phenotype during embryonic vascular development than the full knockout mice. However, we found that DUSP3-deficiency prevented neo-vascularisation of Matrigel plugs and LLC xenograft tumors suggesting that DUSP3 plays an important and non-redundant function in tumor-induced angiogenesis.

Using microarray analysis, we evaluated the expression levels of all DUSPs transcripts in the Matrigel plugs extracts retrieved form DUSP3^−/−^ and WT mice. We found that among all DUSPs (typical and atypical), DUSP1/MKP1 and DUSP23/VHZ were significantly downregulated in Matrigel plugs retrieved RNAs (Table 2) and we did not observe an increase in any DUSP in the absence of DUSP3. Altered expression of DUSP1/MKP1 has been reported in different human cancer (reviewed in [28]). In angiogenesis, DUSP1/MKP1 expression is associated with increased invasiveness of NSCLC due to an increased expression of VEGFC, suggesting that DUSP1 inhibition could be a good strategy to inhibit tumor invasion and angiogenesis [29]. Therefore, the observed decrease of neo-angiogenesis in our model could also be due to the decreased DUSP1/MKP1 expression. The other possibility could be that the observed decrease of DUSP1 reflects the decreased number of endothelial and smooth muscle cells in the Matrigels infiltrates (Table 1). As for DUSP23/VHZ, little is known about this phosphatase function. However, DUSP23/VHZ is highly expressed in several human cancers and could play a role in cell cycle regulation [30]. The cellular distribution of DUSP23 is not known. Therefore, it is difficult to conclude if, in our case, the observed decrease of this phosphatase transcript in DUSP3^−/−^ retrieved Matrigels is due to DUSP3 deficiency or reflects the decrease of EC and smooth muscle cells infiltration in Matrigels.

**Table 2:** Dusps genes expression profile from Dusp3-knockout versus WT Matrigel plugs

**Gene name**	**Protein names**	**Ratio *Dusp3*-KO/WT**	***P value***
***Typical Dusps***			
*Dusp1*	MKP1	0.60	0.041*
*Dusp2*	PAC-1	1.0	0.96
*Dusp4*	MKP2	0.48	0.18
*Dusp5*	DUSP5; hVH3; B23	NP	
*Dusp6*	MKP3	1	0.9
*Dusp7*	MKPX	0.9	0.6
*Dusp8*	DUSP8	0.97	0.88
*Dusp9*	MKP4	ND	
*Dusp10*	MKP5	1.5	0.23
*Dusp16*	MKP7	1.2	0.33
*Styxl1*	MK-STYX	ND	
***Atypical Dusps***			
*Epm2a*	Laforin	ND	
*Dusp3*	VHR	ND	
*Dusp11*	PIR1	1	0.75
*Dusp13*	MDSP; TMDP; DUSP13	1.8	0.38
*Dusp14*	MKP6	0.84	0.51
*Dusp15*	VHY	1.1	0.56
*Dusp18*	LMW-DSP20	0.54	0.11
*Dusp319*	SKRP1; LMW-DSP3	1.3	0.057
*Dusp21*	LMW-DSP21	ND	
*Dusp22*	JSP-1; JKAP; MKPX; VHX	1.4	0.078
*Dusp23*	LDP-3; VHZ	0.85	0.04*
*Dusp26*	MKP8, NEAP	1.3	0.71
*Dupd1*	DUPD1	2.5	0.18
*Dusp28*	VHP	1.1	0.31
*Styx*	STYX	1.1	0.43

Data presented in this table are extracted from a genome-wide microarray dataset obtained using Illumina’s multi sample format Mouse WG-6 V2 BeadChip. Data are presented as the ratio of the average values obtained from 2 pools of 3 *Dusp3*-knockout each on 2 pools of 3 WT mice each and the corresponding *p-value*. (*p < 0.05 determined using unpaired student’s *t* test). ND: not detected. NP: no probe/not present on the microarray chip.

In the aortic ring assay, we found that DUSP3 deficiency prevented the sprouting in response to the angiogenic growth factor b-FGF. This finding was further supported by the significant decrease of angiogenic sprouting in the HUVECs spheroid model after *in vitro* downregulation of DUSP3 using RNA interference. Although the underlying mechanism is not clear, these findings suggest that DUSP3 plays an important role in the b-FGF receptor signaling pathways. FGF signalings are involved in a plethora of biological processes leading to: activation of cell proliferation, inhibition of apoptotic signals, activation of cell migration in different cell types and promotion of angiogenesis. FGF activates cell proliferation mainly through the Raf-MEK-ERK MAPK pathway (reviewed in [19]). The fact that DUSP3 dowregulation in HUVECs did not affect cell proliferation and ERK1/2 activation suggests that DUSP3 is dispensable for the b-FGF-induced cell proliferation. We can also exclude the involvement of DUSP3 in FGF PI3K/Akt pro-survival/anti-apoptotic pathway as DUSP3 depletion did not impact HUVECs apoptosis. On the other hand, the phosphorylation of Akt was normally induced in DUSP3-depleted HUVECs. FGF plays also a crucial role in cell migration and angiogenesis. This effect is mediated through the PI3K and PLCγ/PKC activation pathways [19]. We have indeed demonstrated that DUSP3 depletion in HUVECs affected PKC basal and b-FGF induced phosphorylation. PKCs represent a large family of enzyme activated by two secondary messengers, calcium (Ca2+) and diacylglycerol (DAG). Ca2+ increases the affinity of PKC for lipids and DAG induces a high affinity interaction with the membrane leading to its activation [31,32]. To be ready for activation by Ca2+ and DAG, PKC is first phosphorylated by both phosphoinositide-dependent kinase 1 [33] and by autophosphorylation [34]. The autophosphorylation of PKC on serine 660 residue is important for the stability of the enzyme conformation and downstream signal transduction [35,36]. In absence of DUSP3, we found that this autophosphorylation site (Ser660) is hyperphosphorylated, suggesting that PKC is in a ready state to be activated. However, the anti-phospho-PKC Ser660 antibody used detects endogenous levels of several PKC isoforms. To investigate which PKC isoform is affected by DUSP3 depletion, immunoprecipitation of all the isoforms, followed by immunoblotting with phospho-PKC bII Ser660 is required. What is clear so far is that DUSP3 is involved in FGF-induced PKC activation in MAPKs-independent manner. Upon activation with FGF, DUSP3-depleted cells showed a very slight increase in the phosphorylation of the autophosphorylation site of the PKC family proteins compared to the control. This could be due to the fact the hyperactivated status of PKC at basal levels leads to an unresponsive signaling pathway.

We have also investigated if the most recently identified DUSP3 substrate, EGFR in H1299 cells [10], could be affected by DUSP3 depletion in HUVECs. EGFR is an important player in diverse biological processes and is actually targeted by different approaches in various human malignancies [37]. Tyrosine phosphorylation is an important post-translational modification for EGFR-induced signaling after ligand binding. We found that EGFR tyrosine phosphorylation was not affected by DUSP3 deficiency in HUVEC cells neither at basal levels, nor after EGF activation suggesting that DUSP3 is not targeting EGFR in endothelial cells. These results were compatible with recent study where Wagner *et al.* showed that EGFR was not regulated by DUSP3 in the primary NSCLC tumor cells and in the NSCLC cell line H460 [15].

In the DUSP3^−/−^ mice, we also found that the activity of ERK1/2 and JNK1/2 were not affected by DUSP3 deficiency in B cells, T cells, macrophages and platelets (unpublished observations). However, we failed in testing this in mice primary endothelial cells as the purification of sufficient number of these cells without affecting the basal activity of MAPKs was challenging. This is not the first time that previously characterized DUSP substrate specificity is not confirmed in a knockout mice model. Indeed, deficiency of DUSP2/PAC1, a known phosphatase for ERK and p38, does not lead to enhanced ERK and p38 phosphorylation but rather causes an enhanced JNK phosphorylation, suggesting a crosstalk between the different MAPKs that contribute to the observed changes in DUSP2^−/−^ mice [38]. Similarly, knockout of DUSP10/MKP5, a phosphatase known to target p38, does not cause p38 hyperphosphorylation [39]. These inconsistencies are probably due to the use of *in vitro* overexpression/downregulation systems during previous characterizations of DUPS’s substrate specificity, which may not faithfully reflect the outcomes from DUSP-deficient primary cells. Alternatively, the lack of a particular DUSP may be compensated by other DUSPs.

## Conclusions

Taken together, the present study provides evidence for an unexpected physiological role of the dual specificity phosphatase DUSP3 as new key mediator of neovascularization by affecting at least the b-FGF-induced endothelial cell sprouting most probably via the PKC pathway. However, further investigations are required to shed light into the role of DUSP3 in angiogenesis and the molecular mechanism in the b-FGF-induced, and perhaps other receptor signaling pathways, involved in angiogenic sprouting.

## Methods

### Generation of DUSP3 knockout mice by disruption of Dusp3 locus

The DUSP3 knockout (KO) mouse was generated by replacing the Exon II with the Neo gene by homologous recombination. A 2.3 Kb fragment containing Exon I and a 4.5 Kb fragment containing the 5’ region of Intron II of the *Dusp3* gene were cloned inside the plasmid pPNT and the plasmid was transfected into the 129/SvJ embryonic stem (ES) cell line by electroporation. G418 and Ganciclovir resistant ES clones were screened by PCR using a forward primer located in the *Dusp3* gene, outside the 2.3 kb fragment cloned in the plasmid, and a reverse primer located in the Neo gene. The proper homologous recombination was verified by Southern hybridization analysis, detecting an additional 4.5 Kb fragment after *XbaI* digestion and hybridization with a probe located in the 5’ region of the *Dusp3* gene. Two recombinant ES cell lines were injected into blastocysts of C57BL/6 mice producing chimeras that were mated with C57BL/6 mice to generate heterozygous founders. ES transfection and blastocyst injection were performed at the Moores Cancer Center/Transgenic and Gene Targeting core facility at UCSD. http://cancer.ucsd.edu/Research/Shared/tgm/default2.asp. Heterozygous mice were mated to generate +/+ and −/− littermates to be used for experimentation. Mice were weaned and ear-marked at day 21. At week 4, 2 mm of tail was cut for genotyping using a surgical blade. Total DNA was extracted from tail tip using High Pure PCR template preparation kit (Roche, Vilvoorde, Belgium) and 0.1 μg was used as a template in 50 μl of a final reaction mixture which contained the *Dusp3* primers 5′GTGTGAGCTGCACTTTCCAA3′ and 5′GGTGACTGGGTGAAGAATGG3′, together with the Neo primer 5′TTGCCAAGTTCTAATTCCATCAGA3′. The reaction generates a 456 bp fragment from the *Dusp3* gene and a 365 bp fragment from the recombinant construct.

### Ethical statement

All mice experiments and procedures were carried out following the guidelines and in agreement with the animal ethics committee of the University of Liège. All the work was covered by the ethical licence: 858 “understanding the role of DUSP3 in angiogenesis”.

### Antibodies and reagents

Anti-Von Willebrand Factor (vWF) antibody was from Dako (Dako, Heverlee, Belgium). Anti-DUSP3 antibody used for immunohistochemistry, basic-Fibroblast growth factor (b-FGF) and heparin were from R&D (R&D Systems, Minneapolis, MN). Anti-DUSP3 antibody used for western blots, as well as the anti-CD31, Matrigel and Dispase were from BD Biosciences (BD Biosciences, San Jose, CA). Anti-phosphotyrosine antibody (4G10) was from Millipore (Millipore, Overijse, Belgium). Anti-phospho-ERK1/2 (Thr202/Tyr204), anti-ERK1/2, anti-cJun, anti-pospho-Akt (Ser473), anti-Akt, anti-EGFR, anti-phospho-PKC pan (Ser660) antibodies and SAPK/JNK kinase assay kit were all from Cell Signaling (Cell Signaling, Danvers, MA). HRP conjugated anti-mouse and anti-rabbit secondary antibodies and enhanced chemiluminescence kit (ECL) were from GE Healthcare (GE Healthcare Europe GmbH, Diegem, Belgium). Double stranded siRNA used as a non-targeting control (siCTL) was from Dharmacon (Thermo Scientific-Dharmacon, Erembodegem-Aalst, Belgium). Double stranded siRNAs used for DUSP3 silencing were from Eurogentec (Eurogentec, Seraing, Belgium) and sequences were siDUSP3-1 (GGCAGAAGAUGGACGUCAA), siDUSP3-2 (GGUCCUUCAUGCACGUCAA). Anti-rat Alexa 594 secondary antibody were from Life Technology (Life Technology, Gent, Belgium). Gene*Trans*II was from Mo Bi Tec (Mo Bi Tec, Gottingen, Germany). [3H] thymidine was from Perkin Elmer (Perkin Elmer, Zaventem, Belgium). Anti-GAPDH antibody and Fluorescein isothiocyanate-dextran (FITC-Dextran) were from Sigma (Sigma-Aldrich, Diegem, Belgium). Collagen R was from Serva (Serva, Heidelberg, Germany).

### Cell culture and siRNA transfection and cellular proliferation

Human Umbilical Vein Endothelial Cells (HUVEC), EBM medium and EGM Singlequot were purchased from Lonza (Lonza, Basel, Switzerland). HUVECs were maintained in EGM (EBM + EGM Singlequot). Early cell passages (2 to 6) were transfected with non-targeting siCTL (150nM) or with 2 different DUSP3 targeting siRNA (150nM) using Gene*Trans*II transfection reagent as a vehicle (3.5 μL/1 mL). HUVEC were used for experiments 72 hours after transfection. Cellular proliferation was measured as previously reported [11,17]. The Lewis Lung Carcinoma (LLC) cells were cultured in DMEM (Lonza, Basel, Switzerland) supplemented with 10% heat-inactivated fetal bovine serum in a humidified atmosphere of 5% CO2 at 37°C.

### Immunohistochemistry

Human cervix carcinoma paraffin embedded serial sections (4 μm) were incubated during one hour in the oven at 60°C, deparaffinized and rehydrated using successive baths as follow: 2 × 5 min in xylol, 2 × 2 min in 100% ethanol, 1 × 1 min in 95% ethanol, 1 × 2 min in 70% ethanol and 2 × 2 min in dH_2_O. Antigen retrieval was performed using Target retrieval solution (Dako) for 40 min at 99°C. After 20 min at room temperature (RT), endogenous peroxydases were inhibited using Peroxydase blocking solution (Dako) during 10 min at RT. Background staining was reduced by incubating the slides in 10% normal goat serum/PBS for 30 min at RT. Sections were then subsequently incubated with the primary anti-DUSP3 (dilution: 1/50) or anti-vWF (dilution: 1/200) antibodies for 1 h at RT then with the HRP conjugated anti-mouse or anti-rabbit secondary antibody at 1/200 dilution during 1 h at RT. Staining was revealed using 3,3’-Diaminobenzidine (DAB) chromogen and slides were counterstained with haematoxylin.

### Tubulogenesis Matrigel assay

To perform tube formation assay, 200 μL of Matrigel were put in 24 well culture plates and incubated for 2 hour at 37°C to allow gelling. Dissociated (3 × 10^3^) HUVECs were diluted in the appropriate medium and added onto the Matrigel layer. After 24 h, tube formation was visualized using phase-contrast microscopy. Total tube length and number of intersections were quantified using Image J software (National Institutes of Health, Bethesda, MD).

### Time-lapse video microscopy

siCTL and siDUSP3 transfected HUVECs (10^5^ cells) were seeded on gellified Matrigel layer in 2 wells Lab-Tek chamber slides (Thermo Fisher Scientific, Waltham, MA, USA) and transferred to the stage of a Nikon A1R microscope (Nikon, Wavre, Belgium) equipped with x, y and z axes and maintained at 37°C and 12 hours. Images were acquired every 10 minutes using Nis Elements software (Nikon, Wavre, Belgium) and saved as ND2 files. Individual files were then combined and processed into AVI Movies using Nis Elements software. Representative snap shots were taken from siCTL and siDUSP3 conditions at different time intervals.

### *In vivo* Matrigel angiogenesis assay and LLC cells injection

DUSP3^+/+^ and DUSP3^−/−^ mice were subcutaneously injected in the two flanks with 500 μl of Matrigel supplemented with b-FGF (250 ng/ml) and Heparin (0.0138 mg/ml). Ten days later, Matrigel plugs were carefully harvested, weighted and digested with Dispase for 1 h at 37°C. The hemoglobin content was determined by a colorimetric assay using Drabkin’s reagent (Sigma-Aldrich). In separated experiments, 5 min prior mice sacrifice, freshly prepared FITC-Dextran (100 mg/kg) was injected in the tail vein. Matrigel plugs were frozen in Tissue-tek for subsequent immuno-fluorescence analysis.

For LLC tumor cells injection, mice were subcutaneously injected in the flanks with 10^6^ LLC cells. Seven days later, tumors were carefully harvested, weighted and mechanically grounded using a homogenizer. The hemoglobin content was determined using Drabkin’s reagent colorimetric assay.

### Immunofluorescence staining

For immunofluorescent staining of frozen Matrigel plugs, sections of 7 μm were fixed in ice-cold acetone for 2 min then in methanol (4°C) for 5 min. After blocking in PBS containing 10% normal goat serum for 30 min at RT, slides were incubated for one hour with anti-CD31. Slides were then washed. Immunoreactivity was revealed using anti-rat Alexa 594 secondary antibody. CD31 staining and injected FITC-dextran fluorescence were visualized under Olympus Vanox AHBT3 epifluorescent microscope (Olympus, Aartselaar, Belgium). The number of CD31^+^ blood vessels sections and total FITC-Dextran fluorescence intensity were quantified using Imaris software (Imaris, Bitplane, Zurich, Switzerland).

### Mouse aortic ring assay

Mouse aortic ring assay was performed as previously described [40]. Briefly, 1 mm long mice aortic rings explants were cultured in collagen gel (1,5 mg/ml). The aortic rings were either non-stimulated, stimulated with autologous serum or stimulated with 20 ng/ml of b-FGF. The explants were cultured for 9 Days at 37°C and 5% C02 and photographed using Zeiss Axiovert 25 (Zeiss, Zaventem, Belgium). Microvessel intersections number and maximal length of vessels outgrowth were quantified with the Aphelion 3.2 software from Adsis (Meythet, France).

### Spheroid sprouting assay

To generate the spheroids, we proceeded as previously reported [41]. Briefly, HUVECs resuspended in EBM containing 0.24% high viscosity methyl cellulose (Sigma-Aldrich) were seeded in 96 well round bottom non-adherent plates and cultured overnight at 37°C. Each spheroid contained 10^3^ cells. Single spheroids were collected, embedded in rat tail collagen type 1 gel (Corning, Seneffe, Belgium) and cultured for 48 hours at 37°C in 2% FBS supplemented EBM with 75 ng/ml phorbol-12 myristate 13-acetate (PMA) or 10 ng/ml b-FGF. To quantify the sprouting, the mean number of sprout in each condition was counted.

### Cell lysates, immunoprecipitation, western blot and SAPK/JNK Kinase assay

For western blot experiments, cells were stimulated for the indicated time points and lysed using RIPA buffer (50 mMTris-HCl (pH = 8.0), 150 mM NaCl, 1% NP-40, 0.5% sodium deoxycholate, 0.1% SDS, 1 mM orthovanadate, complete protease inhibitor cocktail tablets EDTA free and 1 mM phenylmethylsulfonyl fluoride) on ice during 20 minutes. Lysates were next clarified by centrifugation at 21000 g during 20 min at 4°C. The resulting supernatants were collected and protein concentrations were determined using the colorimetric Bradford reagent (Bio-Rad, Nazareth, Belgium). Samples were next denaturated at 95°C in Laemmli buffer. To investigate the SAPK/JNK activity, we used the SAPK/JNK kinase assay kit following the instructions of the manufacturer. Briefly, cells were stimulated for the indicated time and lysed with the cell lysis buffer provided. Cell lysates were incubated overnight at 4°C with Phospho-SAPK/JNK Rabbit mAb sepharose beads with constant agitation. Kinase assay was performed by adding c-Jun recombinant protein and ATP to the beads with 1× Kinase buffer and incubated for 30 min at 30°C. The reaction was stopped by adding SDS Laemmli buffer and boiled at 95°C for 5 min. Samples were then run on SDS-PAGE gel and transferred to Hybond-nitrocellulose membranes. To block the non-specific binding sites, membranes were incubated for one hour in Tris-buffered saline-Tween 20 containing 5% of non fat milk or 3% BSA. Membranes were next incubated with anti-Phospho-c-Jun and anti-c-Jun. Immunoprecipitations of EGFR were carried out following previously reported protocols [42]. To evaluate the efficiency of siRNA transfection, the phosphorylation of ERK1/2 and Akt, cell lysates from transfected endothelial cells were resolved by SDS-PAGE and transferred onto nitrocellulose membranes. The membranes were next immunoblotted with anti-DUSP3, anti-phospho-ERK1/2 and anti-phospho-Akt antibodies. Membranes were next stripped, blocked and immunoblotted with anti-GAPDH, anti-Akt and anti-ERK1/2 antibodies for normalization. Immunoreactivity was then revealed using HRP conjugated secondary antibodies. The blots were developed by enhanced chemiluminescence (Amersham, Gent, Belgium) according to the manufacturer’s instructions.

### Microarray analysis and gene expression profiles

Total RNA was isolated from b-FGF containing Matrigel plugs retrieved from DUSP3^+/+^ and DUSP3^−/−^ mice 10 days after sub-cutaneous injection. RNA was prepared using Trizol reagent (Roche). The yield of the extracted RNA was determined using spectrophotometer by measuring the optical density at 260 nm. The purity and quality of the extracted RNA were evaluated using the Experion RNA StdSens Analysis kit (Bio-Rad Laboratories, Hercules, CA). High quality RNA with RNA Quality Indicator (RQI) score greater than 8 was used for microarray experiment. Gene expression profiling was performed using Illumina’s multi-sample format Mouse WG-6 V2 BeadChip containing 45281 transcripts and profiles six samples simultaneously on a single chip (Illumina Inc., San Diego, CA). For each sample, 250 ng of total RNA was labeled using Illumina Total Prep RNA Amplification kit (Ambion, Austin, TX) according to the manufacturer’s instructions. Briefly, double stranded cDNA was synthesized using T7-oligo (dT) primers and followed by an *in vitro* transcription reaction to amplify antisense RNA (aRNA), while biotin was incorporated into the synthesized aRNA probe. The aRNA probe was then purified and quantified using a NanoDrop spectrophotometer (Thermo Fisher Scientific, Waltham, MA).

Biotinylated cRNA probe was hybridized to the Mouse WG-6 V2 BeadChip Array (Illumina). Labeled aRNA (1500 ng) was used for hybridization to each array. The hybridization, washing and scanning, were performed according to the manufacturer’s instructions. The arrays were scanned using a BeadArray Reader (Illumina). The microarray images were registered and extracted automatically during the scan according to the manufacturer’s default settings. Raw microarray intensity data were analysed with the Genome Studio software normalized using the quantile normalization method according to the manufacturer’s recommendation. The probes were considered as expressed by filtering data on Detection *p-value* lower than 0.05. Data are presented as the ratio of the average values obtained from 2 separate pools of Matrigels retrieved from 3 DUSP3^−/−^ mice on 2 separate pools of Matrigels retrieved from 3 DUSP3^+/+^ mice and the corresponding *p-value* was determined using unpaired student’s *t* test. A value of *p* < 0.05 was considered as statistically significant.

### Statistical analysis

The student *t*-test was used to assess statistical differences between different groups. Results were considered as significant if p-value < 0.05. Results are presented as mean ± SEM. Prism software (GraphPad, San Diego, CA) was used to perform statistical analysis. * = p < 0.05, ** = p < 0.01, *** = p < 0.001.

## Competing interests

The authors declare that they have no competing interests.

## Authors’ contributions

AM performed, western blotting, proliferation assays, immunohistochemistry, siRNA transfection, *in vitro* tubulogenesis, *in vivo* Matrigel plug and aortic ring assays. CE helped for the aortic ring and the spheroid assays and for the *in vivo* Matrigel assay. BK helped for the *in vivo* Matrigel plug assays and edited the manuscript. FC made the cloning to generate DUSP3 knockout mouse. BS helped for the quantification of tubulogenesis, spheroid and the aortic rings sprouting. MM and FD helped for live imaging assay. SP and VM helped for the mice handling, cell transfection and western blotting. TZ performed the LLC xenograft tumor assay in mice. PD helped for mice breeding and handling. TM supervised and provided the grant support for the generation of the DUSP3 knockout mouse. MM and LM participated in discussion of the results and edited the manuscript. NA and GC participated in design of the experiments and discussion and edited the manuscript. SR designed and supervised the study, drafted the manuscript and provided grant support for this study. All authors read and approved the final version of this manuscript.

## Supplementary Material

Additional file 1 DUSP3 downregulation affects HUVEC cells angiogenic sprouting. 72 hours after transfection with siCTL, HUVECs were trypsinized and 7.5 × 10^3^ from each transfection condition were seeded on Matrigel solidified matrix in Lab-Tek chamber slides. Chambers were immediately transferred to the stage of a Nikon A1R microscope and maintained for 16 hours at 37°C and 5% CO2 culture conditions. Images were acquired every 10 minutes using Nis Elements software and saved as ND2 files. Individual files were then combined and processed into AVI. Representative movie for siCTL (movie 1) condition are shown. Click here for file

Additional file 2 DUSP3 downregulation affects HUVEC cells angiogenic sprouting. 72 hours after transfection with siDUSP3-1, HUVECs were trypsinized and 7.5 × 10^3^ from each transfection condition were seeded on Matrigel solidified matrix in Lab-Tek chamber slides. Chambers were immediately transferred to the stage of a Nikon A1R microscope and maintained for 16 hours at 37°C and 5% CO2 culture conditions. Images were acquired every 10 minutes using Nis Elements software and saved as ND2 files. Individual files were then combined and processed into AVI. Representative movie for siDUSP3 (movie 2) condition are shown. Click here for file

## References

[B1] Tautz L Critton DA Grotegut S Protein tyrosine phosphatases: structure, function, and implication in human disease Methods Mol Biol 2013 1053 179 221 10.1007/978-1-62703-562-0_13 23860656 PMC8158066

[B2] Patterson KI Brummer T O’Brien PM Daly RJ Dual-specificity phosphatases: critical regulators with diverse cellular targets Biochem J 2009 418 475 489 19228121 10.1042/bj20082234

[B3] Dhillon AS Hagan S Rath O Kolch W MAP kinase signalling pathways in cancer Oncogene 2007 26 3279 3290 10.1038/sj.onc.1210421 17496922

[B4] Lawrence MC Jivan A Shao C Duan L Goad D Zaganjor E Osborne J McGlynn K Stippec S Earnest S Chen W Cobb MH The roles of MAPKs in disease Cell Res 2008 18 436 442 10.1038/cr.2008.37 18347614

[B5] Kim EK Choi EJ Pathological roles of MAPK signaling pathways in human diseases Biochim Biophys Acta 2010 1802 396 405 10.1016/j.bbadis.2009.12.009 20079433

[B6] Nunes-Xavier C Roma-Mateo C Rios P Tarrega C Cejudo-Marin R Tabernero L Pulido R Dual-specificity MAP kinase phosphatases as targets of cancer treatment Anticancer Agents Med Chem 2011 11 109 132 10.2174/187152011794941190 21288197

[B7] Ishibashi T Bottaro DP Chan A Miki T Aaronson SA Expression cloning of a human dual-specificity phosphatase Proc Natl Acad Sci U S A 1992 89 12170 12174 10.1073/pnas.89.24.12170 1281549 PMC50720

[B8] Yuvaniyama J Denu JM Dixon JE Saper MA Crystal structure of the dual specificity protein phosphatase VHR Science 1996 272 1328 1331 10.1126/science.272.5266.1328 8650541

[B9] Alonso A Saxena M Williams S Mustelin T Inhibitory role for dual specificity phosphatase VHR in T cell antigen receptor and CD28-induced Erk and Jnk activation J Biol Chem 2001 276 4766 4771 10.1074/jbc.M006497200 11085983

[B10] Wang JY Yeh CL Chou HC Yang CH Fu YN Chen YT Cheng HW Huang CY Liu HP Huang SF Chen YR Vaccinia H1-related phosphatase is a phosphatase of ErbB receptors and is down-regulated in non-small cell lung cancer J Biol Chem 2011 286 10177 10184 10.1074/jbc.M110.163295 21262974 PMC3060470

[B11] Rahmouni S Cerignoli F Alonso A Tsutji T Henkens R Zhu C Louis-dit-Sully C Moutschen M Jiang W Mustelin T Loss of the VHR dual-specific phosphatase causes cell-cycle arrest and senescence Nat Cell Biol 2006 8 524 531 10.1038/ncb1398 16604064

[B12] Cerignoli F Rahmouni S Ronai Z Mustelin T Regulation of MAP kinases by the VHR dual-specific phosphatase: implications for cell growth and differentiation Cell Cycle 2006 5 2210 2215 10.4161/cc.5.19.3267 17012840

[B13] Henkens R Delvenne P Arafa M Moutschen M Zeddou M Tautz L Boniver J Mustelin T Rahmouni S Cervix carcinoma is associated with an up-regulation and nuclear localization of the dual-specificity protein phosphatase VHR BMC Cancer 2008 8 147 10.1186/1471-2407-8-147 18505570 PMC2413255

[B14] Arnoldussen YJ Lorenzo PI Pretorius ME Waehre H Risberg B Maelandsmo GM Danielsen HE Saatcioglu F The mitogen-activated protein kinase phosphatase vaccinia H1-related protein inhibits apoptosis in prostate cancer cells and is overexpressed in prostate cancer Cancer Res 2008 68 9255 9264 10.1158/0008-5472.CAN-08-1224 19010898

[B15] Wagner KW Alam H Dhar SS Giri U Li N Wei Y Giri D Cascone T Kim JH Ye Y Multani AS Chan CH Erez B Saigal B Chung J Lin HK Wu X Hung MC Heymach JV Lee MG KDM2A promotes lung tumorigenesis by epigenetically enhancing ERK1/2 signaling J Clin Invest 2013 123 12 5231 5246 10.1172/JCI68642 24200691 PMC3859406

[B16] Hao L ElShamy WM BRCA1-IRIS activates cyclin D1 expression in breast cancer cells by downregulating the JNK phosphatase DUSP3/VHR Int J Cancer 2007 121 39 46 10.1002/ijc.22597 17278098

[B17] Wu S Vossius S Rahmouni S Miletic AV Vang T Vazquez-Rodriguez J Cerignoli F Arimura Y Williams S Hayes T Moutschen M Vasile S Pellecchia M Mustelin T Tautz L Multidentate small-molecule inhibitors of vaccinia H1-related (VHR) phosphatase decrease proliferation of cervix cancer cells J Med Chem 2009 52 6716 6723 10.1021/jm901016k 19888758 PMC2790023

[B18] Alonso A Rahmouni S Williams S van Stipdonk M Jaroszewski L Godzik A Abraham RT Schoenberger SP Mustelin T Tyrosine phosphorylation of VHR phosphatase by ZAP-70 Nat Immunol 2003 4 44 48 10.1038/ni856 12447358

[B19] Turner N Grose R Fibroblast growth factor signalling: from development to cancer Nat Rev Cancer 2010 10 116 129 10.1038/nrc2780 20094046

[B20] El Hour M Moncada-Pazos A Blacher S Masset A Cal S Berndt S Detilleux J Host L Obaya AJ Maillard C Foidart JM Ectors F Noel A Lopez-Otin C Higher sensitivity of Adamts12-deficient mice to tumor growth and angiogenesis Oncogene 2010 29 3025 3032 10.1038/onc.2010.49 20208563

[B21] Berndt S Perrier d’Hauterive S Blacher S Pequeux C Lorquet S Munaut C Applanat M Herve MA Lamande N Corvol P van den Brule F Frankenne F Poutanen M Huhtaniemi I Geenen V Noel A Foidart JM Angiogenic activity of human chorionic gonadotropin through LH receptor activation on endothelial and epithelial cells of the endometrium Faseb J 2006 20 2630 2632 10.1096/fj.06-5885fje 17065221

[B22] Berndt S Blacher S Munaut C Detilleux J Perrier d’Hauterive S Huhtaniemi I Evain-Brion D Noel A Fournier T Foidart JM Hyperglycosylated human chorionic gonadotropin stimulates angiogenesis through TGF-beta receptor activation Faseb J 2013 27 1309 1321 10.1096/fj.12-213686 23233533

[B23] Carmi Y Voronov E Dotan S Lahat N Rahat MA Fogel M Huszar M White MR Dinarello CA Apte RN The role of macrophage-derived IL-1 in induction and maintenance of angiogenesis J Immunol 2009 183 4705 4714 10.4049/jimmunol.0901511 19752225

[B24] Blacher S Devy L Burbridge MF Roland G Tucker G Noel A Foidart JM Improved quantification of angiogenesis in the rat aortic ring assay Angiogenesis 2001 4 133 142 10.1023/A:1012251229631 11806245

[B25] Wishart MJ Dixon JE The archetype STYX/dead-phosphatase complexes with a spermatid mRNA-binding protein and is essential for normal sperm production Proc Natl Acad Sci U S A 2002 99 2112 2117 10.1073/pnas.251686198 11842224 PMC122327

[B26] Yang CY Li JP Chiu LL Lan JL Chen DY Chuang HC Huang CY Tan TH Dual-specificity phosphatase 14 (DUSP14/MKP6) negatively regulates TCR signaling by inhibiting TAB1 activation J Immunol 2014 192 1547 1557 10.4049/jimmunol.1300989 24403530

[B27] Ganesh S Delgado-Escueta AV Sakamoto T Avila MR Machado-Salas J Hoshii Y Akagi T Gomi H Suzuki T Amano K Agarwala KL Hasegawa Y Bai DS Ishihara T Hashikawa T Itohara S Cornford EM Niki H Yamakawa K Targeted disruption of the Epm2a gene causes formation of Lafora inclusion bodies, neurodegeneration, ataxia, myoclonus epilepsy and impaired behavioral response in mice Hum Mol Genet 2002 11 1251 1262 10.1093/hmg/11.11.1251 12019206

[B28] Wancket LM Frazier WJ Liu Y Mitogen-activated protein kinase phosphatase (MKP)-1 in immunology, physiology, and disease Life Sci 2012 90 237 248 10.1016/j.lfs.2011.11.017 22197448 PMC3465723

[B29] Moncho-Amor V Ibanez De Caceres I Bandres E Martinez-Poveda B Orgaz JL Sanchez-Perez I Zazo S Rovira A Albanell J Jimenez B Rojo F Belda-Iniesta C Garcia-Foncillas J Perona R DUSP1/MKP1 promotes angiogenesis, invasion and metastasis in non-small-cell lung cancer Oncogene 2011 30 668 678 10.1038/onc.2010.449 20890299

[B30] Tang JP Tan CP Li J Siddique MM Guo K Chan SW Park JE Tay WN Huang ZY Li WC Chen J Zeng Q VHZ is a novel centrosomal phosphatase associated with cell growth and human primary cancers Mol Cancer 2010 9 128 10.1186/1476-4598-9-128 20509867 PMC2893100

[B31] Newton AC Protein kinase C: structure, function, and regulation J Biol Chem 1995 270 28495 28498 10.1074/jbc.270.48.28495 7499357

[B32] Newton AC Protein kinase C. Seeing two domains Curr Biol 1995 5 973 976 10.1016/S0960-9822(95)00191-6 8542286

[B33] Le Good JA Ziegler WH Parekh DB Alessi DR Cohen P Parker PJ Protein kinase C isotypes controlled by phosphoinositide 3-kinase through the protein kinase PDK1 Science 1998 281 2042 2045 9748166 10.1126/science.281.5385.2042

[B34] Keranen LM Dutil EM Newton AC Protein kinase C is regulated *in vivo* by three functionally distinct phosphorylations Curr Biol 1995 5 1394 1403 10.1016/S0960-9822(95)00277-6 8749392

[B35] Edwards AS Newton AC Phosphorylation at conserved carboxyl-terminal hydrophobic motif regulates the catalytic and regulatory domains of protein kinase C J Biol Chem 1997 272 18382 18390 10.1074/jbc.272.29.18382 9218480

[B36] Bornancin F Parker PJ Phosphorylation of protein kinase C-alpha on serine 657 controls the accumulation of active enzyme and contributes to its phosphatase-resistant state J Biol Chem 1997 272 3544 3549 10.1074/jbc.272.6.3544 9013603

[B37] Seshacharyulu P Ponnusamy MP Haridas D Jain M Ganti AK Batra SK Targeting the EGFR signaling pathway in cancer therapy Expert Opin Ther Targets 2012 16 15 31 10.1517/14728222.2011.648617 22239438 PMC3291787

[B38] Jeffrey KL Brummer T Rolph MS Liu SM Callejas NA Grumont RJ Gillieron C Mackay F Grey S Camps M Rommel C Gerondakis SD Mackay CR Positive regulation of immune cell function and inflammatory responses by phosphatase PAC-1 Nat Immunol 2006 7 274 283 10.1038/ni1310 16474395

[B39] Zhang Y Blattman JN Kennedy NJ Duong J Nguyen T Wang Y Davis RJ Greenberg PD Flavell RA Dong C Regulation of innate and adaptive immune responses by MAP kinase phosphatase 5 Nature 2004 430 793 797 10.1038/nature02764 15306813

[B40] Masson VV Devy L Grignet-Debrus C Bernt S Bajou K Blacher S Roland G Chang Y Fong T Carmeliet P Foidart JM Noel A Mouse aortic ring assay: a new approach of the molecular genetics of angiogenesis Biol Proced Online 2002 4 24 31 10.1251/bpo30 12734572 PMC145553

[B41] Detry B Erpicum C Paupert J Blacher S Maillard C Bruyere F Pendeville H Remacle T Lambert V Balsat C Ormenese S Lamaye F Janssens E Moons L Cataldo D Kridelka F Carmeliet P Thiry M Foidart JM Struman I Noel A Matrix metalloproteinase-2 governs lymphatic vessel formation as an interstitial collagenase Blood 2012 119 5048 5056 10.1182/blood-2011-12-400267 22490679

[B42] Rahmouni S Vang T Alonso A Williams S van Stipdonk M Soncini C Moutschen M Schoenberger SP Mustelin T Removal of C-terminal SRC kinase from the immune synapse by a new binding protein Mol Cell Biol 2005 25 2227 2241 10.1128/MCB.25.6.2227-2241.2005 15743820 PMC1061611

